# Beyond mutations: Accounting for quantitative changes in the analysis of protein evolution

**DOI:** 10.1016/j.csbj.2024.06.017

**Published:** 2024-06-21

**Authors:** Xiaoyong Wu, Shesh N. Rai, Georg F. Weber

**Affiliations:** a Biostatistics and Informatics Shared Resources, University of Cincinnati Cancer Center, College of Medicine, Cincinnati, OH, USA; b Cancer Data Science Center, University of Cincinnati College of Medicine Department of Biostatistics, Health Informatice and Data Sciences, Cincinnati, OH, USA; c University of Cincinnati Cancer Center, College of Pharmacy, Cincinnati, OH, USA

**Keywords:** Protein sequence, Phylogenetic tree, Wavelet analysis, Matrix distance, Heat map, Clustering

## Abstract

Molecular phylogenetic research has relied on the analysis of the coding sequences by genes or of the amino acid sequences by the encoded proteins. Enumerating the numbers of mismatches, being indicators of mutation, has been central to pertinent algorithms. Specific amino acids possess quantifiable characteristics that enable the conversion from “words” (strings of letters denoting amino acids or bases) to “waves” (strings of quantitative values representing the physico-chemical properties) or to matrices (coordinates representing the positions in a comprehensive property space). The application of such numerical representations to evolutionary analysis takes into account not only the occurrence of mutations but also their properties as influences that drive speciation, because selective pressures favor certain mutations over others, and this predilection is represented in the characteristics of the incorporated amino acids (it is not born out solely by the mismatches). Besides being more discriminating sources for tree-generating algorithms than match/mismatch, the number strings can be examined for overall similarity with average mutual information, autocorrelation, and fractal dimension. Bivariate wavelet analysis aids in distinguishing hypermutable versus conserved domains of the protein. The matrix depiction is readily subjected to comparisons of distances, and it allows the generation of heat maps or graphs. This analysis preserves the accepted taxa order where tree construction with standard approaches yields conflicting results (for the protein S100A6). It also aids hypothesis generation about the origin of mitochondrial proteins. These analytical algorithms have been automated in R and are applicable to various processes that are describable in matrix format.

## 1 Introduction

### 1.1 Mutation, selection and self-organization

Our contemporary understanding of evolution is largely based on the investigations of genetically encoded biomolecules. In the examination of molecular evolution, the evaluation of coding sequences or amino acid sequences for proteins represents a central tool. Whereas evolution is directed by mutation [8] and selection [7], the latter being constrained by self-organization [18], the molecular analysis of the history of life has struggled with how to account for all three underlying forces.

Mostly, the discussion has focused on tree construction consecutive to the analysis of sequence mismatches. To estimate proper branch lengths, various methods have been explored, including distance-, parsimony-, and maximum likelihood-based [25], [33]. Bayesian inference and modeling algorithms have been applied [15], [20]. In general, the reliability of model-based phylogenetic inference methods is limited by the adequacy of the models that are assumed to approximate the evolutionary process [6]. Improved evolutionary analysis via modifications on the input side of evaluating sequence differences have not found much attention.

**Fig. 1:**
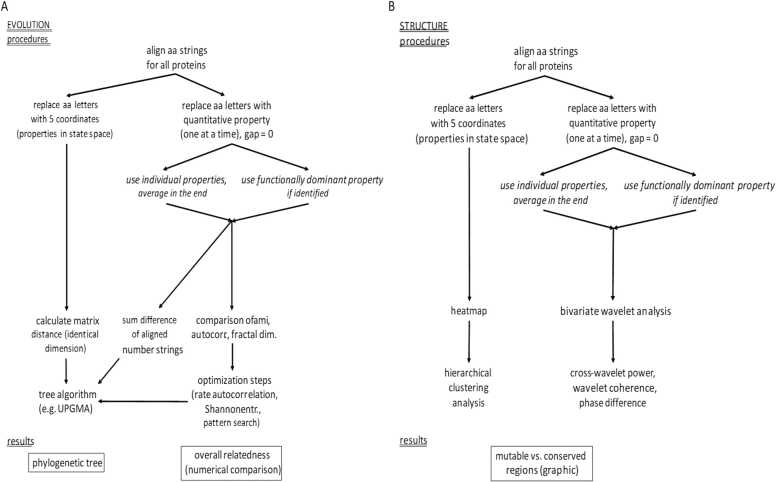
Flow chart for quantitative phylogenetic analysis. Amino acid strings are converted to numerical descriptors. Those enable evolutionary analysis **(A)** as well as structural comparisons **(B)**. In the construction of phylogenetic trees, new input algorithms become available for the calculation of distances. Additionally, numerical comparison for overall relatedness among proteins is feasible, which is inaccessible on the basis of letter strings. The novel structural comparisons comprise heatmaps and bivariate wavelet analyses, both of which result in graphic depictions of mutable versus conserved regions. aa = amino acid, autocorr. = autocorrelation, ami = average mutual information, fractal dim. = fractal dimension, Shannon entr. = Shannon entropy, UPGMA = unweighted pair group method with arithmetic mean.

The conceptual basis for using amino acid mismatches in evolutionary studies of proteins is provided by the mechanism of DNA mutation, which changes individual bases in the coding sequence and then effectuates adaptations of the expressed amino acids. Substitution models of evolution describe the process of genetic variation through fixed mutations and constitute a basis of the evolutionary analysis on the molecular level [2]. Yet, the focus on the manifestation of amino acid changes (or amino acid frequencies [29] does not account for the circumstance that selection and self-organization favor certain mutations over others (natural selection could have acted in sequence space to select non-random sequences satisfying the requirement for a large energy gap between native and unfolded conformations [23]. Notably, such accounting has become principally feasible over the past years, in which a substantial body of knowledge has been accrued to describe the physical and chemical properties of amino acids. These data enable a higher level of discrimination in the comparison of protein sequences through moving the investigation from mismatches to quantitative differences among the mutated and selected amino acids. By incorporating such readouts, previously inaccessible analytical techniques become available. The evolutionary distances, computed from quantitative descriptors, likely have better precision than the distance estimates resulting from currently practiced methodologies (Fig. 1).

### 1.2 From amino acid letters to physico-chemical properties

Empirical numbers have been collected for all 20 amino acids that are utilized by higher species (and are expandable to those 22, which appear in the genetic code of all life [24]), to differentiate their individual physical or chemical characteristics (Supplemental Fig. S1). These measurements entail good estimates for the quantitative description of relevant biochemical features. Further, multiple such properties can be combined in a state space [28], in which each amino acid is described by a unique set of coordinates (Supplemental Table S1) that are useable in phylogenetic analysis. The replacement of the letter codes, which can only distinguish matches or mismatches of the amino acids, with measured magnitudes of their features [30] produces strings of numbers or matrices of coordinates that are amenable to investigation with the algorithms of complex systems analysis. From those, numerical estimations are accessible for the pairwise relatedness between those strings of numbers (including autocorrelation, average mutual information, and fractal dimension) or among those vector space positions (matrix distance, clustering). Subsequently, phylogenetic trees can thus be assembled that are more quantifiable.

• For the taxa under study, the sequences may be aligned, followed by replacing all amino acids with the quantitative descriptor for the physical or chemical property of interest and replacing gaps with 0 (which yields number strings of identical lengths for all taxa). Although the precise values describing the molecular characteristics of the isolated amino acids may undergo some skewing after their integration into a protein as compared to the free amino acids, they embody good approximations for the quantitative description of the molecular properties. This method generates as many analyses as there are properties under consideration. Averaging over all results obtained is a viable strategy.
• Pertaining to specific proteins, selected physico-chemical properties can be more critical for function than others (such as the importance of solubility/hydropathy for membrane proteins, the isoelectric points for regulators of hypoxia, and the likes). When such characteristics are known from operational studies, it is a reasonable presumption that their evaluation offers a more correct approximation of the evolutionary history than features of lesser operational significance. Furthermore, computations of mutual information can aid in evaluating the relative advantages among diverse algorithms by appraising the significance values of particular alignments [22].
• While the conversion from letter strings to number strings constitutes progress, it allows for the analysis of only one property at a time. To consider the amino acid characteristics as comprehensively as possible in one singular examination, the eigenvectors and eigenvalues of a 5-dimensional property space represent a good starting point (the covered properties are normalized with mean value of 0 and standard deviation of 1). For gaps, all coordinates are 0. The 5-dimensional state space was arrived at through principal component analysis, which tends to assign higher weight to outliers. This may be beneficial to evolutionary analysis, where changes between or among sequences are the focus of the investigation. While this approach accounts for properties overall, it requires an entirely new set of tools compared to strings of letters or of numbers. A suitable description conceptualizes matrices for each taxon/clade under study, which comprise the number of positions in the protein (including gaps) x 5 state space coordinates describing the properties of each amino acid (row by row). Pairwise comparisons are feasible to assess the similarity or distance between the matrices thus assembled. Once having produced all pairwise comparisons, one can apply dendrogram-generation algorithms to construct a tree from the calculated distances.

Amino acids possess measurable characteristics. Our prior work [30] has supported the hypothesis that the conversion of protein sequences from the constituent amino acid letters to quantitative values renders the proteins amenable to quantitative assessment of their similarities. Here, we take the analysis to the next level with the conversion to matrices, which represent coordinates in a state space, comprehensively describing biochemical features. The distances calculated from the numerical (not just Boolean) input data yield phylogenetic trees that display more consistency than traditional algorithms, and they agree well with biological expectations. Besides, overall similarity and mutable regions can be studied separately. When compared to the conventional approach, the benefit of the matrix-based evaluation is most tangible for the study of divergent sequences.

## 2 Methods

### 2.1 Sequence conversion

The conversion of amino acid letter strings to numbers that represent individual properties is done according to Supplemental Fig. S1 [30]. The assignment of eigenvectors and eigenvalues in 5-dimensional space is outlined in Supplemental Table S1 [28].

### 2.2 Autocorrelation and average mutual information

The autocorrelation statistic assesses the extent of linear dependence between number strings (waves). The degree of similarity is reflected in the size of its dimensionless number. The formula for autocorrelation is composed of terms for variance and autocovariance. The calculated coefficients range from − 1 to + 1, with + 1 indicating perfect synchrony and − 1 reflecting exact mirror images. The total lack of correlation yields 0.

The average mutual information is defined as MI = H(X) + H(Y) - H(X,Y), where H represents the marginal or the joint entropy function, with X and Y standing for the input strings. Here, it was computed with the mi.empirical function in the R package entropy, wherein the Shannon entropy is calculated.

### 2.3 Bivariate wavelet analysis

The wavelet analysis was carried out using the R package WaveletComp (version 1.1). The Morlet wavelet (composed of a complex exponential – the carrier, multiplied by a Gaussian window – the envelope) constitutes the basis of the package in analyzing the frequency structure of bivariate series. The Morlet mother wavelet used has the form

(1) $$\psi \left(t\right)=\pi^{-\frac{1}{4}}\exp\left(i\omega t-\frac{t^{2}}{2}\right)$$

where ω is the angular frequency or rotation rate in radians per progression unit. The preferred value of it is 6, which leads to an approximately analytic form of the Morlet wavelet. An alternative application is wavethresh, which gives flexibility in the selection of parent wavelets.

### 2.4 Matrix distances

We briefly describe notations for the matrix distances. Suppose that there are several datafiles under investigation and in each datafile, there are n species and m positions are present in each species, where the m positions in a species consists of combination of 20 amino acids and missing value (replaced by zero). Each position (amino acids) is summarized by 5 numerical descriptors called E1-E5 [28] (Supplemental Table S1) and these are non-zero values; the E1-E5 for a missing amino acid are all zero.

A variation on the Euclidean distance can be calculated for the amino acid at each position and then summed up for the amino acid at all positions. Let the variable $x_{\mathrm{ijk}}$ denote numerical measure of the k^th^ descriptor at the j^th^ position for the i^th^ species and for i=1,...,n,j=1,…,m,k=1,…,5. The distance between the amino acids at the j^th^ position for the i ^th^ and the $i^{\prime \mathrm{th}}$ species is given

(2) $$d_{ii^{\prime }}^{\left(j\right)}=\sqrt{\sum_{k=1}^{5}{\left(x_{\mathrm{ijk}}-x_{i^{\prime }\mathrm{jk}}\right)}^{2}};\;i,i^{\prime }=1,...,n;,j=1,\ldots ,m$$

This is summed up over all amino acids at all positions to yield the variation on the Euclidean distance between the i^th^ species and the $i^{\prime th}$ species

(3) $$d_{ii^{\prime }}=\sum_{j=1}^{m}d_{ii^{\prime }}^{\left(j\right)}=\sum_{j=1}^{m}\sqrt{\sum_{k=1}^{5}{\left(x_{\mathrm{ijk}}-x_{i^{\prime }\mathrm{jk}}\right)}^{2}};i,i^{\prime }=1,...,n.\;$$

Alternatively, Frobenius distances were calculated using the FDist2() function in R (v.4.1.2) package SMFilter (v.1.0.3), which defined the Frobenius distance between the i^th^ species and the i′^th^ species as

(4), $$d\left(X_{i},X_{i^{\prime }}\right)=\;\sqrt{\mathrm{tr}\left\{{\left(X_{i}-X_{i^{\prime }}\right)}^{\tau }\left(X_{i}-X_{i^{\prime }}\right)\right\}},i,i^{\prime }=1,\ldots ,n$$

where $X_{i}=\left[\left(x_{\mathrm{ijk}}\right)\right]$ and$\;X_{i^{\prime }}=\left[\left(x_{i^{\prime }\mathrm{jk}}\right)\right]$ are matrices of size m x 5 each.

It is obvious to see that

(5) $$\mathrm{tr}\left\{{\left(X_{i}-X_{i^{\prime }}\right)}^{\tau }\left(X_{i}-X_{i^{\prime }}\right)\right\}=\mathrm{tr}\left\{{\left(X_{i}-X_{i^{\prime }}\right)\left(X_{i}-X_{i^{\prime }}\right)}^{\tau }\right\}=\sum_{j=1}^{m}\sum_{k=1}^{5}{\left(x_{\mathrm{ijk}}-x_{i^{\prime }\mathrm{jk}}\right)}^{2}i,i^{\prime }=1,\ldots ,n$$

Thus, the result based on the Frobenius distance between the i^th^ species and the i′^th^ species is

(6) $$d\left(X_{i},X_{i^{\prime }}\right)=\;\sqrt{\sum_{j=1}^{m}\sum_{k=1}^{5}{\left(x_{\mathrm{ijk}}-x_{i^{\prime }\mathrm{jk}}\right)}^{2}}$$

Note that there is a difference between (3), (6), but we mostly use equation (3) in in the final analysis. We have automated the process of matrix generation and distance calculations in R, starting from sequences, which have been aligned in Clustal Omega [https://www.ebi.ac.uk/Tools/msa/clustalo/] and are saved as txt files. The R routine (patent pending) strips redundant information, reassembles the individual strings, and converts them into matrices using the values from Supplemental Table S1 for each amino acid and assigning the coordinates 0, 0, 0, 0, 0 to gaps (and to unknown amino acids where applicable). For all pairs of matrices, it then calculates the Euclidean distances. Ensuing to the obtainment of the matrix distances, a tree-generating algorithm is executed; for which existing code was available through the functions ape and phangorn (Supplemental Fig. S2).

## 3 Results

### 3.1 Comparison of similarity among proteins

**Fig. 2:**
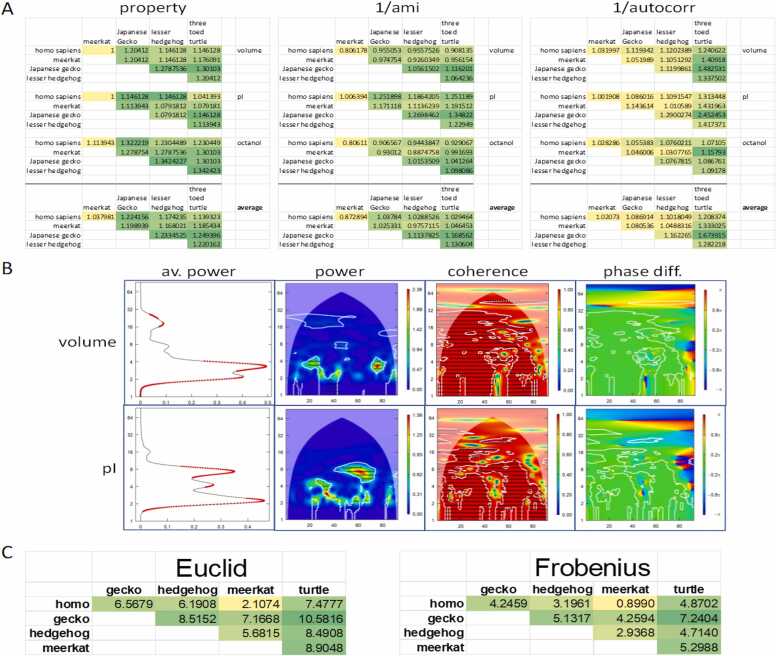
Overall similarity among S100A6 proteins. Wavelet analysis for individual properties across five species. **A)** For volume, isoelectric point (pI), octanol partition coefficient (octanol), and their combined average, the physico-chemical properties (property), the inverse of the average mutual information (1/ami), and the inverse of the autocorrelation (1/autocorr) were compared pairwise among all sequences. The color coding displays the lowest values as yellow and the highest values as green. **B)** Bivariate wavelet analysis was conducted for the comparison between Homo sapiens and the three-toed turtle. For the properties of volume and isoelectric point, displayed are the plots for the average power (av. power), the cross-wavelet power, the coherence, and the phase difference (phase diff.). **C)** Distances between matrix descriptions of the proteins were compared pairwise among all sequences. The color coding displays the lowest values as yellow and the highest values as green.

To compare relatedness or similarity between proteins, it is desirable to assign to each protein under investigation one characteristic numeral. Several techniques of complex systems analysis empower quantitative appraisals between strings of numbers. Once suitably converted, the protein sequences under investigation can be examined on the basis of their autocorrelation and average mutual information (harmonic analysis), or their fractal dimension (analysis in conceptual space). These readouts may be averaged across all parameters, or – if justified – a functionally dominant property (such as hydrophobicity or pH) can be selected. Alternatively, after matrix conversion, distances can be calculated. The results provide estimations for phylogenetic relatedness (Fig. 2).

Previously, various computations of mutual information-based distances had generated more discriminating findings than conventional branch lengths in distance-based phylogeny, specifically for a version based on single-letter Shannon entropies [22]. The average mutual information is an information theory quantity that synopsizes a non-linear correlation function, which provides a quantitative readout for the extent of information shared between 2 strings (here the converted sequence data from 2 taxa or species). Reflecting evolutionary relatedness, the average mutual information is calculated by pairwise comparison of protein sequences across all taxa under investigation [30].

The autocorrelation statistic quantifies the degree of linear dependence between proteins, to reflect how much values, originating earlier in development, display some relation to values originating later. It expresses the extent of similarity in a dimensionless number with a magnitude ranging from − 1 to + 1, with + 1 expressing seamless synchrony and − 1 indicating precise mirror images. The formula for autocorrelation entails terms for variance and autocovariance. Expanding evolutionary distance is represented in a reduction of the autocorrelation between 2 data strings (which reflect the physico-chemical parameter under study), calculated pairwise between taxa [30]. Taking this one step further, known patterns of inheritance forecast greater similarity in the tempo of molecular evolution between direct ancestors and descendants than is the case in branched relationships. This connection results in autocorrelation of evolutionary rates. It has been found that rate autocorrelation is a common phenomenon throughout evolution [10], [27].

Fractal geometry has introduced a concept of dimension that offers a numerical readout for the estimation of the geometric complexity by processes or objects, such as 2 quantitative strings signifying distinct species. The fractal markers of dimension are larger than 1 (estimating identity between the taxa and displaying as a data distribution near the 45° angle on a plot that visualizes the numerical values for 2 species against one another) and smaller than 2 (estimating entire independence between the sequences). Whereas distant associations are represented in wider scatter and larger fractal dimensions, smaller box counting dimensions (which estimate the fractal dimension) reflect closer relationships between the numerical strings. Across all pairs of taxa included in the analysis, the box counting dimensions for the amino acid properties by the proteins can be calculated and applied as estimates for their mutual evolutionary distances [30]. The box counting procedure is prone to a strictly positive quantization error that derives from arbitrary grid position and orientation. It causes difficulties for the procedure’s slope estimation step. An efficient means of minimizing the problem is provided by pattern search [3]. Quantitative evolutionary analyses that incorporate the computation of box counting dimensions will benefit from integrating such measures.

Contingent with the focal point of the study, the above features can be investigated individually or for multiple characteristics in conjunction. One can elect to average – across all properties considered – the various values for each readout, average mutual information, autocorrelation, and fractal dimension [30] (see also Fig. 2A). The resulting quantitative results are interpretable as being indicative of evolutionary distance. Hence, the overarching similarity among protein sequences can be numerically appraised by tabulating the values for these mathematical representations.

Overall similarity is also quantifiable between matrices that are composed of the 5 state space coordinates for each amino acid. Through pairwise row-by-row comparisons, a calculated distance (Euclidean, Frobenius or other) yields one numerical value between two matrices (representing the protein of interest between two species) (Fig. 2C). The numbers thus obtained may serve as evolutionary distance estimates and can form the foundation for phylogenetic tree assemblies.

### 3.2 Assembly of phylogenetic trees

The construction of phylogenetic trees is one of the most important methods in evolutionary studies. There are multiple strategies available for application to the assembly of orthodox phylogenetic dendrograms. It is common for conventional trees, which rely on the alignments of amino acids, that they tabulate the frequency of mismatches between aligned sequences as a cornerstone of algorithms that calculate kinship [5], [13]. The underlying paradigm has two main weaknesses. Several such comparisons can be typified by mutually identical frequencies of mismatches and are positioned at identical distance, even though synchronicity in divergence is highly improbable in evolutionary biology. Accounting for gaps is generally problematic and may non-trivially reduce the fraction of residues that are captured by the algorithm (for comparisons among large sets of proteins, this can substantially compromise the investigation [31]).

Both weaknesses associated with these commonly applied tree algorithms, the coverage of gaps and the discrimination among sequences with identical numbers of mismatches, are addressable through the replacement of amino acid letters with quantitative properties. In phylogenetic trees that are derived from numerical quantities, the value 0 can be utilized to account for a gap. As there is a range of values to insert for the individual amino acids (not just 0 or 1 for match or mismatch), the number strings or matrices are highly unlikely to result in the same branch lengths for any pairs of input. Ensuing therefrom, the numeric tree diagrams are more likely than the letter tree diagrams to be a faithful reflection of the true evolutionary distances.

Canonical approaches [21] can serve as a basis for generating phylogenetic dendrograms from the numeric strings. In the example of the unweighted or weighted pair group method with arithmetic mean (UPGMA or WPGMA methods), for each of the properties under investigation, the strings of numbers assigned to the proteins are aligned in pairs, such that all permutations of pairs are included. The absolute of the difference between 2 values in the same position is computed, and the sum of the differences across all positions is added up to produce the sum-difference. In pairwise comparisons across the entire set, the smallest sum-difference between 2 strings of numbers is seen as a quantitative estimate of the evolutionary distance between the closest relatives. With the ensuing step, a combined string is generated for those 2 species with the smallest sum-difference by averaging between the values of the alignment, and the procedure is repeated until all pairwise distances have been computed. The phylogenetic tree is assembled with the branch-lengths reflecting the computed distances. The use of quantitative values for specific properties as a basis, and their comprehensive evaluation in tree generation may map a course to improved approximation of the genuine evolutionary record than the sole dependence on sequence information alone.

Furthermore, the numbers for average mutual information, autocorrelation, and fractal dimension (see description above), calculated between all pairs of proteins in the set to be investigated, are indicative of evolutionary distances and may be mapped in phylogenetic trees. The trees thus generated tend to have close resemblance among each other. Where there are differences, they can be dealt with, in consideration of the underlying biology, either by focusing on functions that are known to be critical for the physiologic role of the molecule or by normalizing and averaging these readouts across all the assigned number strings. Conspicuously, the trees assembled with this innovative, simplified and unified approach have thus far been found by us to be mutually less divergent than the phylogenetic trees produced with a variety of methods that are currently in use for mismatch-based evaluation [30].

The characterization of amino acids in a state space is based on the recognition that their physico-chemical properties within a protein are critical for folding and function, even when the sequence homology is low. Tree generation from the analysis of the matrix description requires an algorithm that compares similarity or distances between matrices. As the axes are unitless and the coordinates are not interdependent, a variation on the Euclidean distance is readily obtained and informative, as it yields one precise numeric value. Another approach is the Frobenius distance that considers the matrix values to represent positions on a Stiefel manifold (additional algorithms are available for determining the distance or similarity between 2-dimensional matrices of identical size – they may offer suitable alternatives; by contrast, the calculation of Euclidean norms, Manhattan norms or simple sums of entries for each matrix individually does not yield suitable quantities to serve as a basis for ensuing calculations of divergence). Once that comparison has been completed pairwise, dendrogram-generating strategies are applicable by utilizing the calculated matrix distances as the distances between the taxa/clades under study.

### 3.3 Refined results of evolutionary analysis

**Fig. 3:**
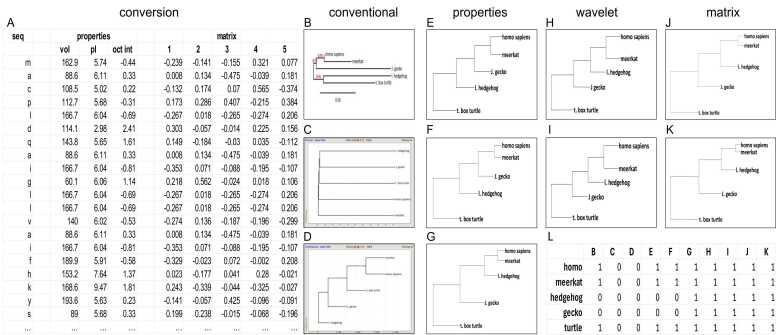
Phylogeny of S100A6. **A)** Conversion of the amino acid sequence to property values to state space coordinates is illustrated on the N-terminal first 20 amino acids of human S100A6. seq = sequence, vol = volume, pI = isoelectric point, oct int = octanol partition coefficient, matrix 1–5 indicates the coordinate values along the 5 state space axes. **B-D)** Conventional algorithms, starting from strings of letters. The internet applications used were phylogeny.fr [http://www.phylogeny.fr/] (B) and two algorithms in Gene Bee [http://www.genebee.msu.su/services/phtree_reduced.html] (C,D). **E-G)** The letter strings were converted to numbers, based on select physico-chemical properties of the individual amino acids. The sum differences were calculated pairwise between species, and the closeness of their values (calculated stepwise after averaging of the two smallest numbers) determined the distances on the trees for octanol partition coefficient (E), volume (F), and isoelectric point (G). **H-I)** For each of the three properties evaluated, average mutual information (H) and autocorrelation (I) were determined pairwise between species. The resultant values were averaged across the three properties. Trees were assembled stepwise from 1/(average mutual information) or 1/autocorrelation, such that the two taxa with the smallest value at each step were combined and their associated numbers were averaged before repeating the process. The positions and distances of the branches in the phylogenetic tree are reflective of the results obtained in the stepwise process. **J-K)** The letter strings were converted to 5-column matrices, based on the amino acid positions in a state space describing their overall properties. The matrix distances were calculated pairwise between species, as Euclidean distance (J) or Frobenius distance (K), and their values provided the input for a stepwise tree generation through combining the closest species, averaging distances, and continuing the process until all distances have been calculated. **L)** True/false table for the phylogenetic trees B) through K). Under the assumption that the ranking of evolutionary development orders the species under analysis as shown in the rows of the table, the value 1 was assigned for trees that matched this expectation, while a value of 0 was entered for different rankings.

Regarding the data input for the assembly of phylogenetic trees, having applied the progression from letter strings via number strings (individual properties, then averages across properties) to matrices has improved the consistency of the outputs. We illustrate the comparison between strings of letters and strings of numbers through the analysis across 5 species of S100A6 (Calcyclin), a short protein with calcium-binding properties (Fig. 3). While most amino acids are identical, there are several mutable residues within the sequence, and there are length differences (gaps) in the C-terminal end. We found conventional trees to be rather divergent (Fig. 3B-D). We replaced the amino acid sequences with the values for octanol interface, volume, and isoelectric point (pI). The trees generated from those numbers with a commonly used method are highly consistent in shape and order (except for the ranks of Japanese gecko versus lesser hedgehog), and they display less difference among them than trees generated with conventional software packages (Fig. 3E-G). The trees calculated from the inverse values of average mutual information or autocorrelation are similar to each other as well as to the trees based on the sum differences of the physico-chemical properties. They also tend to be mutually less divergent than the trees generated with the algorithms currently in use for mismatch-based evaluation. Parenthetically, they appear to resolve the relationships of the lesser hedgehog and the Japanese gecko in favor of a higher ranking for the hedgehog (Fig. 3H-I). The replacement of the amino acid letters with the 5 coordinates in state space for their properties, followed by distance calculation and tree generation, corroborate the higher ranking of the hedgehog (Fig. 3J-K). This is important, because three of the 5 species under study are mammals and two are reptiles (from distinct extant groups), and they should separate accordingly. A clear partition of the mammals from the reptiles aligns with our general understanding of the underlying species relationships, but it is produced only by the more advanced methods (Fig. 3L).

We expanded this proof-of-principle analysis pertaining to the evolution of S100A6 by enrolling a larger number of species. The clustering of known taxa together (as opposed to being broken up into subgroups of branches) was considered to serve as a quality control. While the matrix algorithm is suited to handle gaps (matrix coordinates 0,0,0,0,0), we found that it performs best when their number is kept low – by our estimate below 25 % (Supplemental Fig. S3A). According to the criterion of keeping taxa clustered together, the matrix method produced trees as good (Clustal Omega, Mega 11) or better than (phylogeny.fr) commercial programs, which align on the basis of match/mismatch (Supplemental Figure 3B,C).

**Fig. 4:**
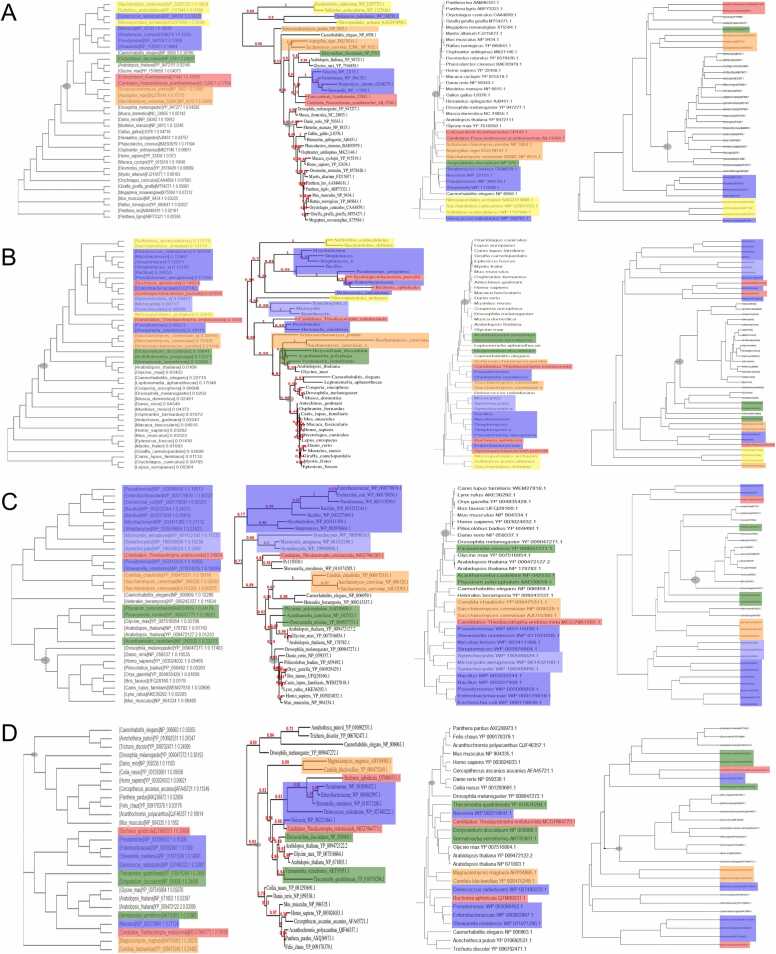
Phylogeny of mitochondrial proteins. Proteins encoded by the mitochondrial DNA were analyzed by conventional algorithms (Clustal Omega with default settings, left; phylogeny.fr with default settings, second from left; Mega 11 with alignment in NCBI Cobalt, saved in Fasta format, Mega align, UPGA method, default settings, third from left) as well as after matrix conversion of the letter strings (right, the UPGMA package was used to construct a phylogenetic tree from a distance matrix computed from the sequence alignment of the species). The proteins for Cytochrome b **(A)**, Cytochrome c oxidase I **(B)**, Cytochrome c oxidase III **(C)** and NADPH Dehydrogenase III **(D)** are displayed. Single-cell organisms are highlighted in blue (bacteria) or light blue (cyanobacteria), yellow (archaea), green (amoebae), orange (yeast) or red (endosymbionts). The nodes delineating the advanced organisms have been manually highlighted. To collect the source sequences, the search started with the NCBI landmark model organisms and then sought to add representatives of diverse clades. Limited N- or C-terminal truncations were implemented to reduce the numbers of gaps in the sequence alignments.

A long-debated topic in evolution is the origin of mitochondria. While it is generally accepted that they were derived from endosymbiosis, the species or taxon that served as the primordial source for mitochondria has not been elucidated [36], [34]. Mitochondria carry their own genetic information, which encodes several proteins [19]. We retrieved sequences for Cytochrome b, Cytochrome c Oxidase I and III, and NADPH Dehydrogenase III from the NCBI landmark model organisms and then sought to add representatives of diverse clades. The matrix analysis consistently finds the higher organisms in proximity to amoebae and yeast (yeast cells lack mitochondria and utilize fermentation to produce energy), whereas three conventional algorithms yield less uniform results (Fig. 4, Supplemental Table S2). The matrix-based study is consistent with amoebae or yeast having been the originators of mitochondria.

We see potential utility for the matrix-based analysis of protein evolution in clinical studies, such as the mutations of proteins in cancer cells due to genetic instability or the mutation of pathogen proteins under the selective pressure of antibiotic treatment. Regarding an example for the latter, the medication of organ transplant patients with mycophenolic acid (which targets inosine 5′-monophosphate dehydrogenase, IMPDH) exerts selective pressure on this enzyme in Pneumocystis jirovecii microorganisms, to which these patients are susceptible. Further, several related fungi have mycophenolic acid-sensitive and -resistant variants [14]. Clustal Omega, Mega 11 and the matrix analysis developed here, all pair IMPDH variants from mycophenolic acid-sensitive and -resistant variants of the same species in close proximity. However, the treatment-induced variants by Pneumocystis jirovecii are grouped on one branch only by Mega 11 and the matrix method, consistent with the biological expectations (Supplemental Fig. S4).

We also tested the matrix-based approach with the analysis of mutations in clinical samples. (Supplemental Fig. S5). In cancer, genomic instability causes an increased frequency of mutations that can affect the coding regions of proteins. During early transformation, the “guardian of the genome” protein TP53 frequently suffers a loss of function in this manner. This, in turn, facilitates a worsening tumor mutational burden (TMB) and enables tumor progression – with regional lymph node infiltration being an early indicator. We retrieved the data for manageably small subsets of breast cancer from cBioportal [https://www.cbioportal.org/], identified their mutant TP53 molecules, and evaluated their phylogenetic analysis with the matrix methods versus three conventional algorithms for their relationships to quantitative clinical readouts. Even for a key molecule in the protection against cancer, a tight correlation with multifactorial clinical readouts cannot be expected. However, there are indications that the quantitative approach to protein mutational analysis developed here is superior to conventional strategies. One can envision that the matrix-based study of several tumor suppressor proteins may lead to additional improvements.

### 3.4 Mutability of domains within proteins

A basic approach to visualizing stable versus unstable regions is the plotting of the values that pertain to the physical or chemical attributes of specific amino acids, displaying the means across species and a readout for their scatter, on a chart of attribute versus position. Regions with large error bars are indicative of evolutionarily unstable domains, whereas regions with small or zero error bars represent domains that are substantially or greatly conserved [30].

Applying a more refined method, the number strings that are reflective of the amino acid features in all positions within a protein may be interpreted as waves (starting at position 1 and extending through the length to the end of the protein). The mathematical techniques of wavelet analysis are well suited to comparing similarities of such waves (see Fig. 2B). In regions that are conserved between two taxa, there is synchrony between the waves that symbolize them, whereas mutated domains display as divergence between the waves. Bivariate wavelet analysis differentiates hypermutable from conserved regions; it is thus able to disclose regions of evolutionary preservation, contrasting from regions of evolutionary instability. Cross-wavelet analysis enables pairwise comparisons from the frequency domain between species, such that a comparison of periodicity between two sequences is illustrated. Possible methods to visualize the results include cross-wavelet power plots, wavelet coherence plots, average power plots, and phase difference images.

• The cross-wavelet average power plot represents a summarized view on the shared periods and the corresponding average power with their attributable statistical significance. Significant joint periods are indicative of low mutability and occur more frequently between species of close evolutionary proximity.
• Whereas the cross-wavelet power resembles covariance, the measure of wavelet coherence is related to correlation, with a range of values from 0 to 1. If 2 sequences have a constant relative phase, they are coherent in periodicity. The coherence plot, being not impacted by wide swings in sequences, is better suited for displaying the areas, wherein 2 sequences jointly share significant periods. Only in regions, where the two series under comparison actually have in common such significant joint periods, does the measure exhibit statistical significance. Coherence is high (canonically displayed in graphs through color shading plus enclosure into contour lines) between species with close evolutionary relationships. The x-coordinates display the mutable domain of the protein, whereas the y-coordinates (Fourier period for synchrony) represent the range of the divergence between taxa. The coherence and cross-wavelet power plots may likewise include arrows to show the region of significant joint periods, with the orientation of the arrows representing the direction of the phase differences.
• In the phase difference image, a global representation is provided for the synchrony between the two variable quantities pertaining to all positions in the amino acid string with differing Fourier periods. The plot shows in-phase relationships between the 2 wavelets as shades of color, such that the amino acid position is represented on the x-axis and the corresponding Fourier period represented on the y-axis. Regions of substantial conservation between the two species under study can be displayed with color shading. Further, the plot displays out-of-phase relationships as various color shades. Those domains are regions within the protein that have experiences evolutionary instability. A pair of waves is deemed coherent if the contributing strings have a constant relative phase.

In the bivariate wavelet analysis, proteins that are close in sequence and have large property differences will tend to match with smaller scales (higher wavelet frequency equates to rapid change) and those that are closer in value will tend to match the larger scales (low frequency equating to slow change). The wavelet transform can be interpreted as a filtering process, where a high value is given to sequence patterns that match the wavelet dynamics. Thus, a good correlation is expected for the patterns that are binned into slower and faster dynamics as dictated by the wavelet kernel and the pattern properties. The choice of the mother wavelet (continuous or discrete; Haar, Daubechies, Morlet or other) will have some impact on the filtering and the resulting covariance. The complex Morlet wavelet can separate the phase and amplitude components within a given signal. A constant phase difference in distinct protein sequences could be interpreted as a travelling wave. This approach allows for dynamical non-stationarity that may arise [12].

**Fig. 5:**
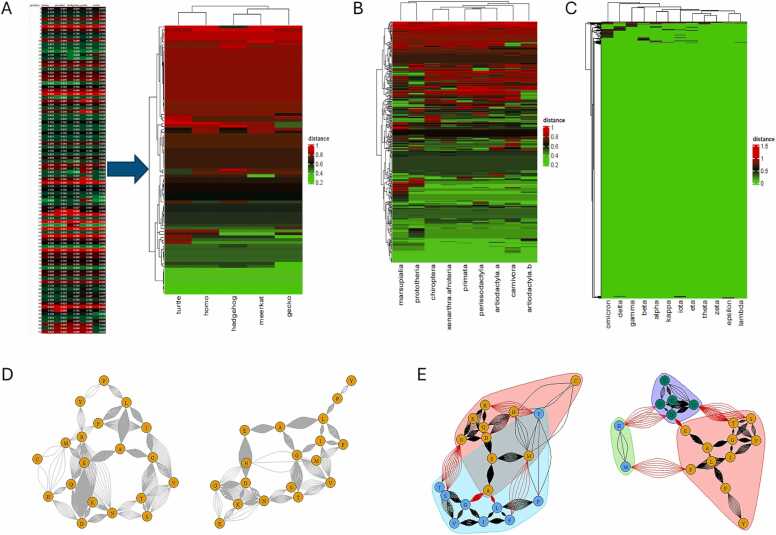
Visualization of regional mutability in the matrix depiction of the amino acid sequences. A-C) Heat maps. **A)** S100A6. For each row (representing amino acids in consecutive positions) the Euclidean distance was calculated to a hypothetical reference matrix that represents the average of the 20 amino acids for each coordinate in the 5-dimensional state space (left). Hierarchical clustering analysis then arranged and put dendrograms on the columns (taxa) and rows (of note, the clustering in this dimension scrambles the amino acid sequence but highlights the extent of differences). **B)** Osteopontin consensus sequences of nine clades. The Euclidean distances were calculated to a hypothetical reference matrix that represents the average of the 20 amino acids for each coordinate in the 5-dimensional state space. Hierarchical clustering rearranged columns and rows. **C)** SARS-CoV-2 Spike Glycoprotein. The reference sequence for calculating Euclidean distances was the reported parent sequence from the start of the COVID-19 pandemic. Hierarchical clustering rearranged columns and rows. **D-E)** Connectivity graphs. S100A6. From the source sequences and alignment in Fig. 3, two species were chosen as examples. R routines were applied to generate the graphs [https://davetang.org/muse/2017/03/16/matrix-to-adjacency-list-in-r/]. The melt() function from the reshape2 package created adjacency lists from the input matrices. Then, igraph generated the display items. In each subfigure, the left graph displays the network of matrix-converted amino acids for the species “Tufted_duck_[XP_032060414.1]; the right graph shows the analogous network for the species ”Human_[AAP36486.1]”. **D)** Network graphs. The igraph package in R was used to display simple graphs. **E)** Newman-Girvan clustering. The igraph package can detect communities or subgraphs (shown in diverse colors) using the Newman-Girvan algorithm.

Regional mutability in the matrix depiction of the amino acid sequences can be visualized through heatmaps. For each row (amino acids in consecutive positions) in the matrices of interest, the Euclidean distance to a reference matrix is calculable. The reference may be a hypothetical “average amino acid” (for each coordinate in the 5-dimensional state space, the average of the 20 actual amino acids is computed) or it may be a parent sequence, such as the originally reported spike glycoprotein sequence in the analysis of named SARS-CoV-2 variants. Hierarchical clustering analysis can aid the evaluation of similarities (Fig. 5A-C). Further, matrices represent d-dimensional data sets of n entries, which are translatable into vectors. They have been visualized in vector and operator spaces, as Markov chains, through harmonics, and in graphs [32]. The conversion and display of amino acid sequences (for the protein under study) via matrices as adjacency graphs visualizes differences in within-protein connectivity between or among species or clades (Fig. 5D,E).

## 4 Discussion

Amino acids possess quantitatively measurable characteristics. The conversion of protein sequences from the constituent amino acid letters to numerical values renders the proteins amenable to mathematical analyses of their similarities (beyond Boolean approaches). Once such sequences have been converted to strings of numbers, the analytical methodologies of complex systems research become pertinent [1] and can produce an abundance of insight. In further improvement, their conversion to matrices, representing coordinates in a 5-dimensional state space, comprehensively describes biochemical features and is amenable to matrix comparisons. In the investigation of non-linear phenomena, there is equivalence between the descriptions of harmonics and conceptual space, across the expression as matrices or as heatmaps or as graphs [32].

• Traditional evolutionary analysis has used the strings of amino acid letters as they are encoded in the genes. The resulting “words” have been analyzed solely for mismatches among the species of interest (assigning 0 for matching letters and 1 for those positions with discrepant letters for the additive generation of sum-differences). The assembly of a phylogenetic tree typically proceeds by pairwise comparisons, averaging the two species closest to each other, renewed pairwise comparison, and so on until the last pair has been calculated. The obtained distances are the branch lengths of the tree.
• Our recent improvement was the replacement of the letters with quantitative values for individual physico-chemical properties. With that, we can analyze the resultant strings as waves and calculate distances not only with a non-Boolean sum-difference method but also on the basis of harmonic analysis (the bivariate wavelet analysis, including average mutual information and autocorrelation) or occupancy of a conceptual space (the fractal dimension).
• The utilization of a vector space to describe the sum of all properties strives to step up yet another level and quantitatively describe the amino acids not by individually measured properties but by an assigned place in a 5-dimensional state space that represents all known properties (we proceed from a string to a matrix). With that representation, different sets of analytical techniques are needed. Matrix distances are readily calculated and can replace the sum differences as input for dendrogram generation. Heatmaps can be assembled though the comparison to a hypothetical or concrete reference sequence and may highlight mutable versus stable regions. Each matrix can be visualized as a graph.

Quantitative evolutionary investigations have the potential to improve the accuracy of phylogenetic studies. Other quantifications are conceivable. A recent study developed a combined random energy model for protein folding and coding under the hypothesis that evolution favors large energy gaps between native and unfolded conformations. These energy levels can potentially be utilized to infer distances [23].

When compared to the conventional approach, the benefit of the matrix-based analysis is most tangible for the study of divergent sequences. This is the case for the mitochondrial proteins (Fig. 4, Supplemental Table S2) and for the cancer-borne P53 (Supplemental Fig. S5). For other proteins, the progression from letter strings via number strings to matrices has also strengthened the consistency of the outputs. We have studied Osteopontin [30], and VEGF [30]. In the comparison of dendrograms, the methodological progression has also improved the agreement with expectations that are based on general biological information about the taxa/clades involved. For very homologous protein sequences, such as the SARS-CoV-2 spike glycoprotein (Supplemental Fig. S6), the benefit of the quantitative matrix-based analysis is incremental (based on adjacency as well as on distance to the expected next neighbor).

The methodology is broadly applicable to comparisons among sequences from individual species (taxa) or among the consensus sequences from higher-order groups (clades), even to mutations in clinical settings. In cancer, genomic instability of the tumor and selective pressure under chemotherapy lead to serial mutations inside the cancer cells over time. The underlying mechanisms have been subject to debate [9], [35]. Likewise, in infectious diseases, serial acquisitions of mutations in the pathogen select for variants that can evade host immunity or antibiotic treatment. Matrix descriptions and distance calculations of the protein sequences affected over time can aid in the reconstruction of the underlying changes. The methods elaborated here are further applicable not just to phylogeny and serial mutations in clinical settings, but to a broad spectrum of phenomena that are subject to change and are numerically describable.

Evolutionary analysis, based on properties rather than letter strings, enables the implicit consideration of all forces of evolution (including selection and self-organization) rather than just mutation. While we are not able to dissect out and separately analyze the three components, our quantitative analysis goes beyond match/mismatch analysis of letters, which only accounted for mutations. Using numerical values to represent amino acid residues in protein sequences is more suitable for protein sequence comparisons and phylogenetic tree construction, because it can encode intrinsic properties of amino acids that are influencing the evolutionary restrictions of proteins in various taxa. Although outside the scope of this report, further developments can be conceived of. With ever increasing capabilities in machine learning, a more comprehensive consideration of physico-chemical characteristics is feasible (the 5-dimensional state space had been derived from 237 properties by principal component analysis [28]. Manifold learning could be applied to the 237 or more properties, and analysis might utilize normalized cross-correlation or Bhattacharyya distance (rather than Euclidean or Frobenius distance). In such a scenario, the most suitable technique for the reduction of dimensionality (i-MAP over t-SNE or PCA) will require evaluation.

Various algorithms have been developed to evaluate and measure distances between proteins. Further study is needed to test how they compare to the matrix distances calculated in the present analysis. The property distance index has been based on the 5 state space coordinates. For two peptides with identical sequences, it is 0, and peptides with conservative substitutions of one or two amino acids have a small property distance value, peptides with a recognizable similarity in their physicochemical properties tend to have property distance indices lower than 10, whereas unrelated peptides have much higher property distance values [16]. The index has been successfully applied for immune activation analysis [17], [26]. While the present study utilizes rooted trees (which imply a hierarchical, branched evolution as the model we embrace), the property distance has been developed for distinct graphical analysis in the online tools DGraph [4]. Other visualizations, distinct from rooted trees, are available through BioLayout [11].

The construction of phylogenetic trees is a key approach in evolutionary studies. The distinct dendrograms generated by diverse methods are interpretable as hypotheses on the developmental paths, which have led to the diversity that exists at the time of analysis. For the accuracy of a phylogenetic tree, there are no final ‘yes’ or ‘no’ answers, just preponderance of evidence in favor of one set of connections versus others (the actual paths are historical and therefore not observable at the time of analysis). The reliability and quantitative accuracy of the input data is critical for the trustworthiness of the output. In this regard, the progression from analyzing letter strings via number strings for individual properties to the matrices of comprehensive descriptors constitutes a substantial refinement of the input data. On the output side, due to multiple available techniques for distance calculation and tree generation, improved quantitative tools for gauging the match to biological expectation are still needed (beyond the true/false assessment in Fig. 3L) to account for multiple and variable representatives within and across clades (such as in Fig. 4).

Stability in evolution is often rooted in the properties of proteins and their constituent amino acids, rather than in sequence identity. In the analysis, however, it may be difficult to decide a priori which of the many properties one should use as readout(s). The availability of various quantitative descriptors, which represent exact property values or a precise spatial relation among all amino acids, has provided new tools for phylogeny research. Their utilization will strengthen the testing of hypotheses and get closer to the truth.

## CRediT authorship contribution statement

**Shesh Rai:** Writing – review & editing, Software, Investigation, Formal analysis. **Georg F. Weber:** Writing – review & editing, Writing – original draft, Formal analysis, Data curation, Conceptualization. **Xiaoyong Wu:** Writing – review & editing, Software, Investigation, Formal analysis, Conceptualization.

## Conflict of Interest

The authors declare no conflict of interest.

## Data Availability

All sequence data were obtained from public databases. Additional information is available upon request. For non-commercial use agreements of the software, please contact the authors.
