# Supplementary material for: Beyond mutations: Accounting for quantitative changes in the analysis of protein evolution
Source: Comput Struct Biotechnol J. 2024 Jun 21;23:2637–47. doi: 10.1016/j.csbj.2024.06.017 (PMC11253266; doi:10.1016/j.csbj.2024.06.017)
Supplement: Figure S1 — Supplementary material [file mmc1.docx]

**Supplement**

**Beyond Mutations: Accounting for Selection and Self-Organization**

**in the Analysis of Protein Evolution**

Xiaoyong Wu, Shesh N. Rai, Georg F. Weber

**Table S1: Components E^1^ to E^5^ of 237 Physico-Chemical Properties for each Amino Acid.** 237 amino acid properties can be approximated by dimension reduction in a 5-dimensional vector space. The numerical descriptors for each amino acid represent coordinates in each dimension of the state space. They are calculated by $\sqrt{\lambda_{\mu}}E_{i}^{\mu}$ for the five eigenvectors $E^{\mu}, \mu=1\ldots5$. $\lambda=$ eigenvalue. The distance between amino acids in identical positions of proteins from distinct sources (taxa or clades) is given by

$$d_{ij}= \sqrt{\sum_{\mu=1}^{5} \left( \sqrt{\lambda}E_{i}^{\mu}-\sqrt{\lambda}E_{j}^{\mu} \right)^{2}}$$

and is then summed up over all amino acids in the protein under investigation. From (Venkatarajan/Braun 2001).

|  |  |  |  |  |  |  | Table S1 |
| --- | --- | --- | --- | --- | --- | --- | --- |
| eigenvector | **E^1^** | **E^2^** | **E^3^** | **E^4^** | **E^5^** |  |  |
| eigenvalue | 1961.504 | 788.200 | 539.776 | 276.624 | 244.106 |  |  |
| **A** | 0.008 | 0.134 | –0.475 | –0.039 | 0.181 |  |  |
| **R** | 0.171 | –0.361 | 0.107 | –0.258 | –0.364 |  |  |
| **N** | 0.255 | 0.038 | 0.117 | 0.118 | –0.055 |  |  |
| **D** | 0.303 | –0.057 | –0.014 | 0.225 | 0.156 |  |  |
| **C** | –0.132 | 0.174 | 0.070 | 0.565 | –0.374 |  |  |
| **Q** | 0.149 | –0.184 | –0.030 | 0.035 | –0.112 |  |  |
| **E** | 0.221 | –0.280 | –0.315 | 0.157 | 0.303 |  |  |
| **G** | 0.218 | 0.562 | –0.024 | 0.018 | 0.106 |  |  |
| **H** | 0.023 | –0.177 | 0.041 | 0.280 | –0.021 |  |  |
| **I** | –0.353 | 0.071 | –0.088 | –0.195 | –0.107 |  |  |
| **L** | –0.267 | 0.018 | –0.265 | –0.274 | 0.206 |  |  |
| **K** | 0.243 | –0.339 | –0.044 | –0.325 | –0.027 |  |  |
| **M** | –0.239 | –0.141 | –0.155 | 0.321 | 0.077 |  |  |
| **F** | –0.329 | –0.023 | 0.072 | –0.002 | 0.208 |  |  |
| **P** | 0.173 | 0.286 | 0.407 | –0.215 | 0.384 |  |  |
| **S** | 0.199 | 0.238 | –0.015 | –0.068 | –0.196 |  |  |
| **T** | 0.068 | 0.147 | –0.015 | –0.132 | –0.274 |  |  |
| **W** | –0.296 | –0.186 | 0.389 | 0.083 | 0.297 |  |  |
| **Y** | –0.141 | –0.057 | 0.425 | –0.096 | –0.091 |  |  |
| **V** | –0.274 | 0.136 | –0.187 | –0.196 | –0.299 |  |  |

**Table S2: Evolutionary Relationships for Mitochondrial Proteins.** For the trees displayed in Figure 4, we counted the nodes from the advanced organisms to the candidate origins of mitochondria. Shown are the distances for Cytochrome b, Cytochromec Oxidase I, Cytochrome c Oxidase III, and NADPH Dehydrogenase III. The color-coded table at the bottom averages the distances from the preceding tables.

**Figure S1: Physico-Chemical Properties of Individual Amino Acids. Top panel)** Table of individual amino acid properties collected from various literature sources. **Lower panels)** Bar graphs comparing the 20 amino acids for various physico-chemical characteristics.

**Figure S2: Flow Chart of Automated Matrix Analysis.** We developed a computer program for the quantitative analysis of processes or structures that are describable by matrices. The input are protein sequences aligned in Clustal Omega. The process has been coded in R. Consecutive to the calculation of all Euclidean distances, a dendrogram-generating routine is executed.

**Figure S3: The phylogeny of S100A6. A)** Sequences were collected from NCBI nucleotide. They entail False_girdled_lizard [XM_053277972.1], Eastern_fence_lizard [XM_042440221.1], Komodo_dragon [XM_044448454.1], Leopard_gecko [XM_054995302.1], Japanese_gecko [XP_015284753.1] (squamata, blue-green), Green_sea_turtle [XM_037883414.2], Three-toed_box_turtle [XP_026513887.1], Diamondback_terrapin [XM_054011113.1], Loggerhead_sea_turtle [XM_048828730.1], Bolson_tortoise [XM_050931051.1], Yellow_pond_turtle [XM_044991384.1], Leatherback_sea_turtle [XM_038383301.2], Painted_turtle [XM_005280618.3], Chinese_pond_turtle [XM_039512971.1] (testudines, dark green), Southern_Grasshopper_Mouse [XP_036046825.1], Mouse [NP_035443.1], Golden_spiny_mouse [XM_051156859.1], Dwarf_hamster [XM_051199163.1], Norwegian_rat [NM_053485.2], Reed_vole [XM_050118676.1], Eastern_gray_squirrel [XM_047515757.1], California_deer_mouse [XM_052730739.1], Alpine_marmot [XM_015502681.2], Bank_vole [XM_048457009.1], Pacific_pocket_mouse [XM_048357994.1], Groundhog [XM_046421839.1], Lesser_Egyptian_jerboa [XM_004667182.2], Banner_tailed_kangaroo_rat [XM_042682208.1], Prairie_deer_mouse [XM_006976234.3], European_water_vole [XM_038311972.1], Creeping_vole [XM_041656281.1], Golden_hamster [XM_005080240.4], White_footed_mouse [XM_028890794.2], Striped_gopher [XM_005331253.4] (rodents, yellow), European_rabbit [NM_001195742.1], Plateau_pika [XM_040998427.1], American_pika [XM_004588925.3] (lagomorpha, green), Lesser_hedgehog [XP_004714789.1], Lesser_hedgehog_tenrec [XM_004714732.2], Masked_shrew [XM_056123855.1], Common_shrew [XM_055140242.1], Spanish_mole [XM_037499347.2], Etruscan_shrew [XM_049782374.1] (eulipotyphla, turcoise), Camelid [XP_006214794.1], Pig [NM_001044557.1], Water_buffalo [XM_055587069.1], Dwarf_musk_deer [XM_055403512.1], Bactrian_camel [XM_010954053.2], Sheep [XM_042243274.1], Blue_whale [XM_036859678.1], Common_warthog [XM_047784575.1], Orca [XM_004284715.4] (artiodactyla, purple), Quagga [XM_046682816.1], Donkey [XM_044758554.1], Wild_horse [NM_001081841.1] (perissodactyla, lilac), Bobcat [XM_047092891.1], Siberian_tiger [XP_007088028.1], Hyena [XP_039074883.1], Racoon_dog [XM_055324259.1], Northern_elephant_seal [XM_045876019.2], Cheetah [XM_027048554.2], Fishing_cat [XM_047840739.1], Eurasian_otter [XM_047705342.1], Leopard [XM_019431597.2], Snow_leopard [XM_049635013.1], Geoffroy_cat [XM_045454264.1], Hawaiian_monk_seal [XM_021682038.2], Dingo [XM_049112655.1], American_black_bear [XM_045784255.1], European_badger [XM_045983001.1], Grey_seal [XM_036066870.1], Arctic_fox [XM_041763934.1], American_mink [XM_044266156.1], Cat [XM_023247824.2], European_polecat [XM_004780393.3], Leopard_cat [XM_043567905.1], Tiger [XM_042976213.1], Lion [XM_042925346.1], Polar_bear [XM_040635662.1], Dog [XM_003434904.3], Jaguarundi [XM_040452921.1], Canada_lynx [XM_030302470.1], California_sea_lion [XM_027614179.2], Southern_elephant_seal [XM_035009599.1] (carnivores, red), Ring_tailed_lemur [XM_045545099.1], Human [AAP36486.1], Pygmy_chimpanzee [XM_003817172.5], Gorilla [XM_004026740.4], Chimpanzee [XM_003308491.4], Sumatran_orangutan [XM_002810156.5], Bornean_orangutan [XM_054453779.1], Common_marmoset [XM_002759980.5], Sunda_slow_loris [XM_053591143.1], Crab_eating_macaque [XM_005541780.2], Tibetan_macaque [XM_050756603.1], Black_capped_squirrel_monkey [XM_010329657.2], Green_monkey [XM_007977085.2], White_faced_capuchin [XM_017513297.1], Rhesus_macaque [NM_001261799.1] (primates, blue). They were analyzed directly (left), after truncation of outlier N-terminal sequences (second from left), after entire removal of sequences that generated large numbers of gaps (second from right), or after further reduction of sequences to be analyzed. The bottom panel indicates the number of positions (amino acids) included, the number of gaps in these positions, and the percentage of gaps. **B,C)** Comparison of three conventional algorithms (Clustal Omega, left and phylogeny.fr, second from left, Mega 11 third from left) to the matrix analysis (right) for the truncated sequence analysis (B) and the most simplified sequence analysis (C).

**
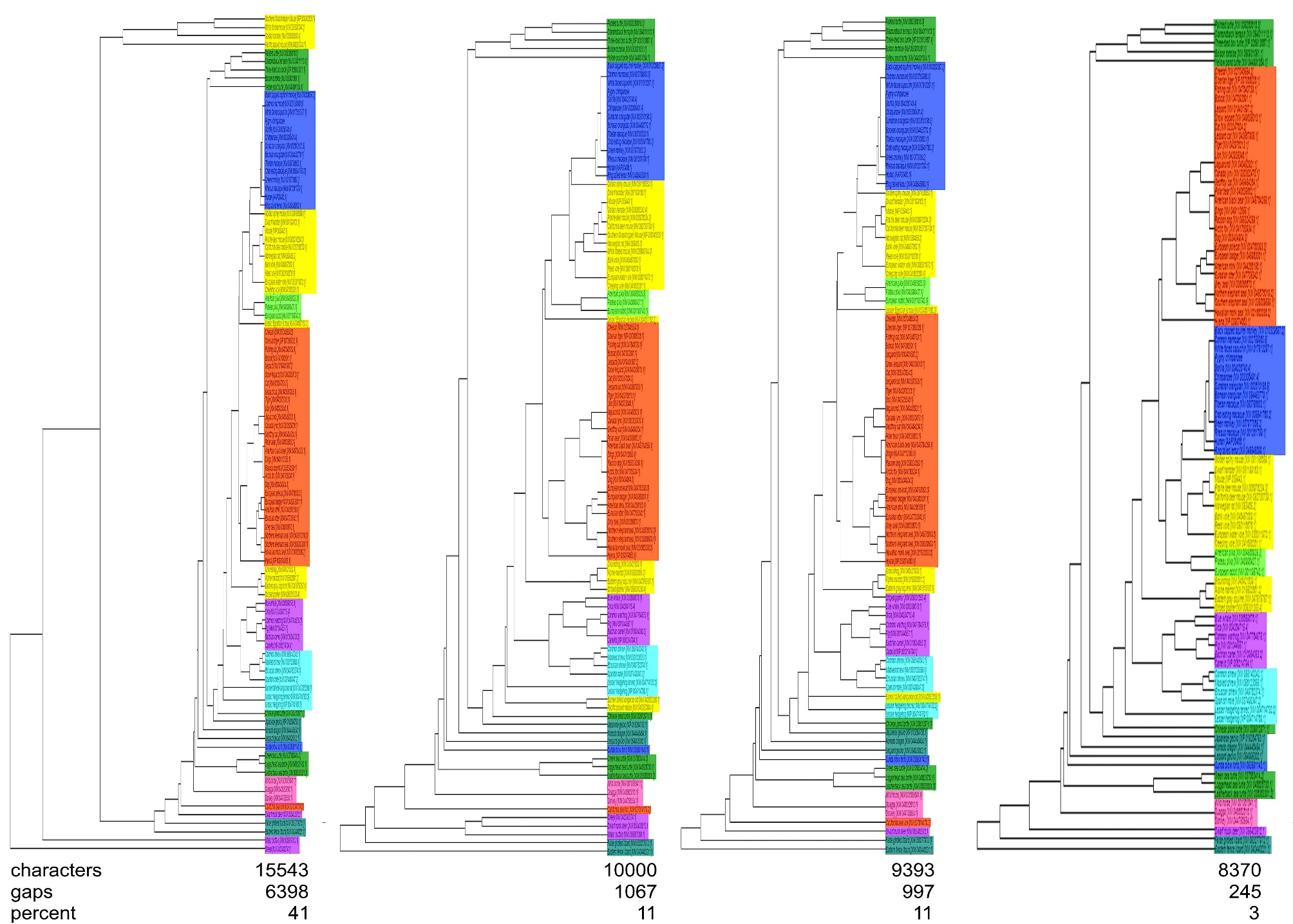
**

**
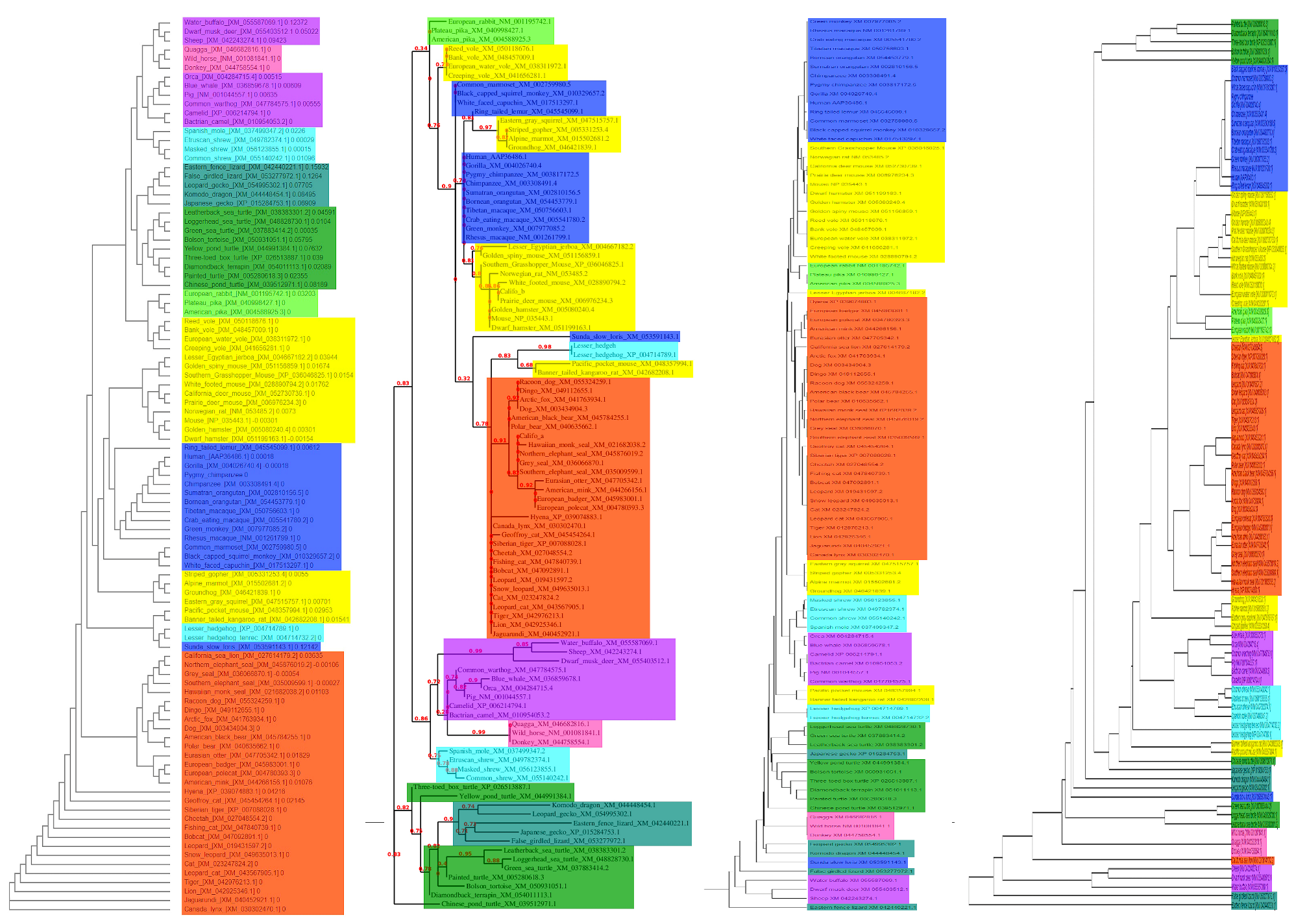
**

**
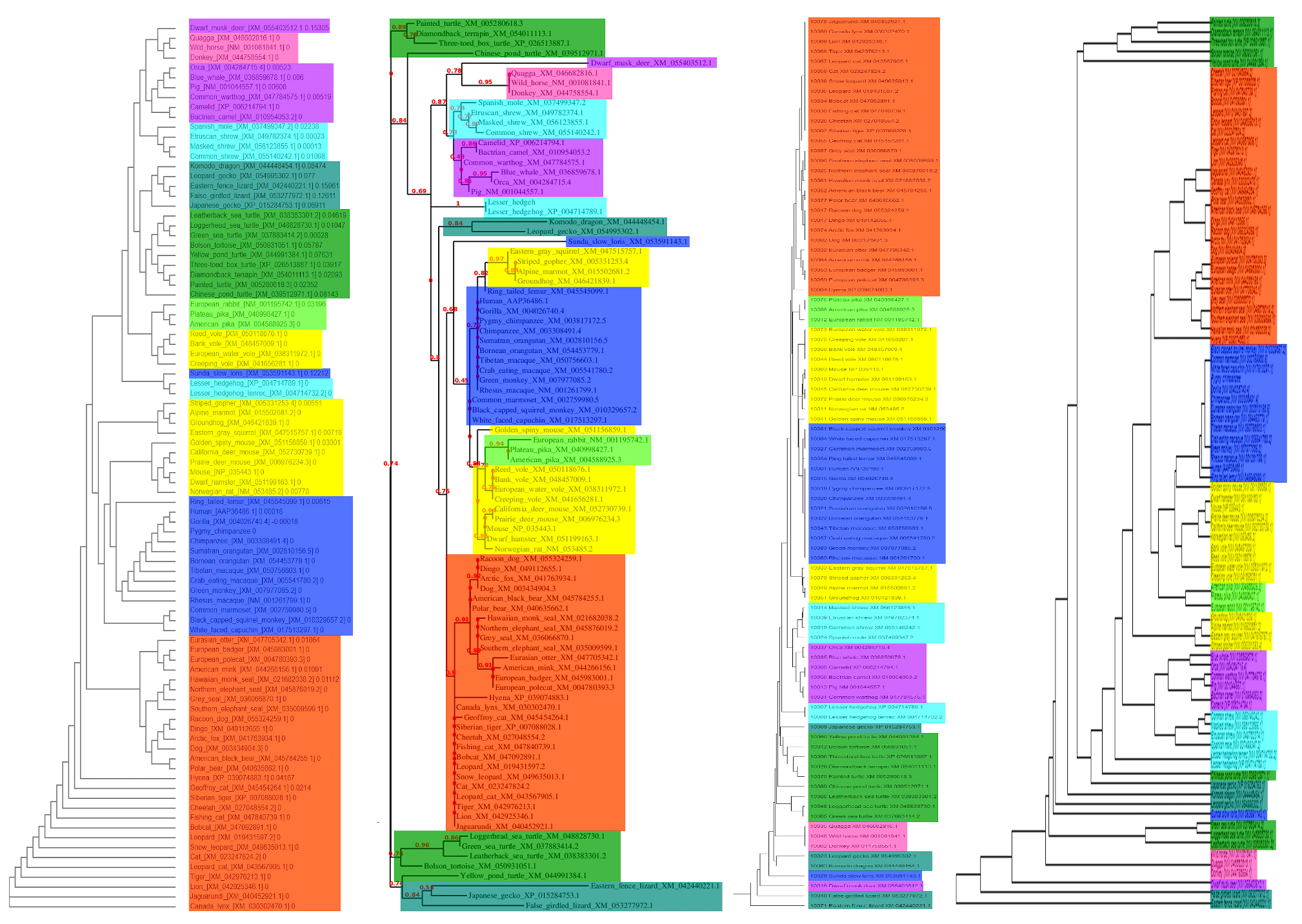
**

**Figure S4: Protein mutations in clinical specimens. Phylogeny of pathogen proteins.** Proteins for fungal Inosine 5’-Monophosphate Dehydrogenase (IMPDH) under the selective pressure of drug treatment with mycophenolic acid were analyzed by applying conventional algorithms (Clustal Omega, left and Mega 11, middle), as well as after matrix conversion of the letter strings (right). PjIMPDH-WT: Pseudomonas jirovecii IMPDH considered wild-type; PjIMPDH-A261T = Pseudomonas jirovecii IMPDH with a missense mutation Ala261Thr; PjIMPDH-A26S: Pseudomonas jirovecii IMPDH with a missense mutation Ala26Ser; PjIMPDH-G439S: Pseudomonas jirovecii IMPDH with a missense mutation Gly439Ser; MgIMPDH1-MPA^S^: MPA-sensitive IMPDH1 of Meyerozyma guilliermondii; MgIMPDH2-MPA^R^: MPA-resistant IMPDH2 of Meyerozyma guilliermondii; ScIMPDH3-MPA^S^: MPA-sensitive IMPDH3 of Saccharomyces cerevisiae; ScIMPDH2-MPA^R^: MPA-resistant IMPDH2 of Saccharomyces cerevisiae; CaIMPDH-MPA^S^: MPA-sensitive IMPDH of Candida albicans; CaIMPDH-MPA^R^: IMPDH of a MPA-resistant strain of Candida albicans; AnIMPDH-MPA^S^: MPA-sensitive IMPDH of Aspergillus nidulans; PbIMPDHA-MPA^R^: MPA-resistant IMPDH-A of Penicillium brevicompactum; PcIMPDH: Pneumocystis carinii IMPDH; PmIMPDH: Pneumocystis murina IMPDH.

**
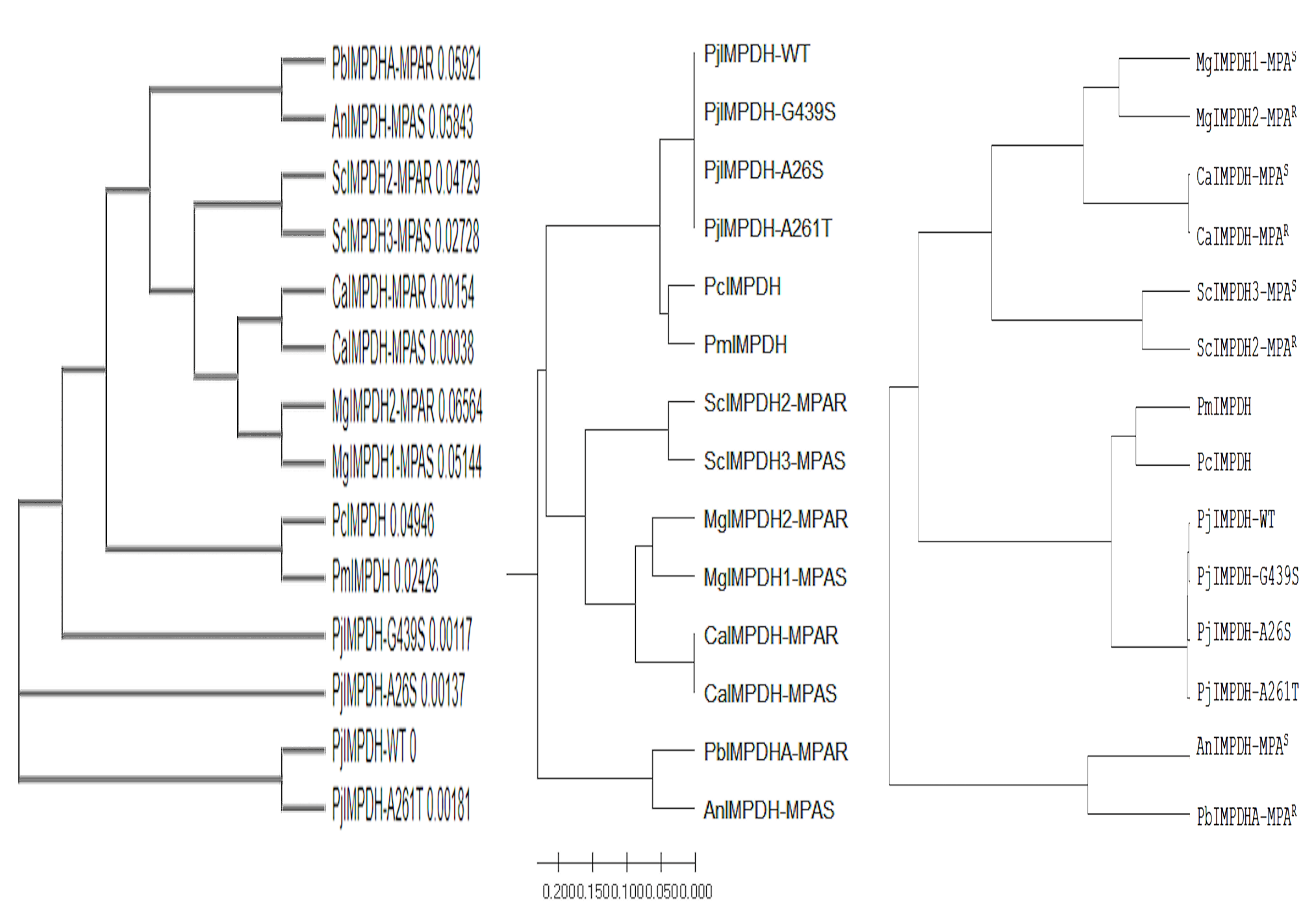
**

**Figure S5: Protein mutations in clinical specimens. TP53 mutations and cancer progression.** Subsets of cancers were analyzed from individual studies in cBioportal. Only missense mutations and in-frame insertions/deletions were included. **(Left panel)** The Metastatic Breast Cancer Project (Provisional, December 2021). Mixed Ductal and Lobular Carcinoma for the readouts of lymph node positivity. **(Middle Panel)** Breast Cancer (METABRIC, Nature 2012 & Nat Commun 2016) Mixed Ductal and Lobular Carcinoma for the readouts of lymph node positivity and tumor mutational burden (TMB, TP53 protects from accumulating mutations). **(Right Panel)** Breast Cancer (METABRIC, Nature 2012 & Nat Commun 2016) Breast Cancer Breast Invasive Lobular Carcinoma for the readouts of lymph node positivity and tumor mutational burden. **A)** The top row displays the matrix-based analysis. **B)** The upper middle row contains the phylogenetic trees generated in Clustal Omega. **C)** The lower middle row shows the results from phylogeny.fr. **D)** The bottom row contains the phylogenetic trees generated in Mega 11 (alignment in NCBI Cobalt, saved in Fasta format, Mega align, UPGA method, default settings). The green separator emphasizes the clustering of lymph node status that is achieved with the matrix method, but not with the two reference algorithms. The yellow highlight indicates an outlier in the matrix clustering, which matches the outlier in tumor mutational burden. The red line separates two distinct groups in Clustal Omega, which do not correspond to clinical distinctions.

**Figure S6: The phylogeny of SARS-CoV-2 Spike Glycoprotein.** Like all retroviruses, SARS-CoV-2 has a high mutation rate due to the absence of genomic repair. The main strains, designated by Greek letters, have individually caused waves during the course of the COVID-19 pandemic. The S1 subunit of the surface structure, Spike Glycoprotein, is particularly important for host interactions, because it binds to cognate receptors on the cell surface. Therefore, its relationships across viral strains may provide information about infectivity, vaccine susceptibility, and pandemic progression. We compare the phylogenetic trees for Spike glycoprotein S1 among strains (Ghosh et al. 2022) according to conventional phylogenetic tree generation and the tree derived from the matrix description.

We analyzed the S1 domain from the main variants. **A)** Conventional algorithm, starting from strings of letters. The internet application used was phylogeny.fr [<http://www.phylogeny.fr/>]. The original output is shown. **B)** Conventional algorithm, starting from strings of letters. The application used was Mega 11 in the default settings, UPGMA method. **C)** Phylogenetic tree based on Euclidean matrix distances for the state space of properties. **D)** Phylogenetic tree based on Frobenius matrix distances for the state space of properties.

# Reference

# Ghosh N., Nandi S., Saha I. A review on evolution of emerging SARS-CoV-2 variants based on spike glycoprotein. Int Immunopharmacol 2022;105:108565.
